# Variables That Best Differentiate In-Patient Acute Stroke from Stroke-Mimics with Acute Neurological Deficits

**DOI:** 10.1155/2016/4393127

**Published:** 2016-12-06

**Authors:** P. Natteru, M. R. Mohebbi, P. George, D. Wisco, J. Gebel, C. R. Newey

**Affiliations:** ^1^Department of Neurology, University of Missouri, Columbia, 5 Hospital Drive, CE 540, Columbia, MO 65211, USA; ^2^Department of Emergency Medicine, University of Missouri, Columbia, Columbia, MO, USA; ^3^Cleveland Clinic, 9500 Euclid Ave, Cleveland, OH 44195, USA; ^4^Akron General Hospital, 3562 Ridge Park Dr, Akron, OH 44333, USA

## Abstract

*Introduction*. Strokes and stroke-mimics have been extensively studied in the emergency department setting. Although in-hospital strokes are less studied in comparison to strokes in the emergency department, they are a source of significant direct and indirect costs. Differentiating in-hospital strokes from stroke-mimics is important. Thus, our study aimed to identify variables that can differentiate in-hospital strokes from stroke-mimics.* Methods*. We present here a retrospective analysis of 93 patients over a one-year period (2009 to 2010), who were evaluated for a concern of in-hospital strokes.* Results*. About two-thirds (57) of these patients were determined to have a stroke, and the remaining (36) were stroke-mimics. Patients with in-hospital strokes were more likely to be obese (*p* = 0.03), have been admitted to the cardiology service (*p* = 0.01), have atrial fibrillation (*p* = 0.03), have a weak hand or hemiparesis (*p* = 0.03), and have a prior history of stroke (*p* = 0.05), whereas, when the consults were called for “altered mental status” but no other deficits (*p* < 0.0001), it is likely a stroke-mimic.* Conclusion*. This study demonstrates that in-hospital strokes are a common occurrence, and knowing the variables can aid in their timely diagnosis and treatment.

## 1. Introduction

Stroke is the fifth leading cause of death in the United States (US) [1]. The economic and personal impact is huge considering the great direct and indirect costs, with a projection of US costs of ischemic stroke hitting $2.2 trillion by 2050 [2]. Intravenous recombinant tissue plasminogen activator (IV rt-PA) is approved for treating strokes in appropriate patients within 4.5 hours of stroke onset [3].

The clinical diagnosis of an acute stroke, however, can be a matter of dispute [4–6]. Typically, an acute stroke is considered in any patient who presents with sudden onset of neurological changes that can be localized to a vascular territory. However, other diagnoses, such as metabolic/infectious encephalopathy, seizure, syncope, peripheral neuropathy, space-occupying lesions, and migraines, may mimic a stroke, as observed in 22 to 31% of patients presenting to the emergency department (ED) with stroke-like symptoms [7, 8]. These may not be recognized until further diagnostic testing is available. In one of the studies by Goyal et al., treating the stroke-mimics (SM) with IV rt-PA led to an excess direct and indirect hospital cost of $257,975 and $152,813, respectively, and the median excess cost per admission was $5401 [9].

Data for in-hospital strokes is, however, limited. This population represents up to 15% of first-time strokes [10]. Although in-hospital stroke-mimics have not been studied as extensively as those presenting to the ED, in-hospital strokes are often more severe and associated with a worse outcome [11]. These observations suggest the importance of recognizing in-hospital strokes and differentiating them from stroke-mimics, which can be difficult in comparison to out-of-hospital strokes.

At our institution, an in-patient acute stroke paging program has been established for over a decade (a.k.a. 2CLOT). It is an alerting system to the stroke fellow, resident, and staff. The purpose of this retrospective data analysis was to evaluate the in-patient stroke consults for the characteristics of strokes and stroke-mimics, that is, the timing, circumstances, and etiologies, with the ultimate goal of identifying differentiating variables that may allow for improved triaging.

## 2. Methods

This study is a retrospective data analysis of consecutive patients over a one-year period (2009 to 2010) at a large, tertiary-care referral center. The local institutional review board (IRB) approved the study.

### 2.1. Inclusion Criteria

The patient sample (18 years and older) included all consecutive acute stroke consults (or what our institution refers to as 2CLOTs), who were already admitted as an in-patient at the time of activation. Additionally, patients admitted to the in-house neurological floor and the neurological intensive care unit were excluded. The acute stroke consult was activated for urgent in-patient evaluations for any change in the neurological status from previous examinations. These consult activations could be from physicians, nurses, or ancillary staff.

### 2.2. Data Collection

Data was retrieved from the electronic medical records. The retrieved data included many variables like the initial National Institute of Health Stroke Scale (NIHSS) score, the circumstances of the brain attacks including time of onset, setting of onset (awakening from sleep, after operation, and sedation), time to presentation/evaluation, and examination at onset (blood pressure and heart rate, current atrial fibrillation, mental status, lateralizing features, neurological examination, and metabolic abnormalities). Historical data included prior medications, surgical history, past medical history (hypertension, ischemic heart disease, tobacco abuse, atrial fibrillation, diabetes mellitus, peripheral vascular disease, and prior stroke), stroke-mimic risk factor history like cognitive impairment, migraine, and epilepsy. Computed tomography (CT) and CT-angiography (CTA) of the head and vessels, magnetic resonance imaging (MRI) brain and magnetic resonance angiography (MRA) of the carotids and the circle of Willis, transcranial Doppler (TCD), electroencephalogram (EEG), and carotid ultrasounds (CUS) were the diagnostic data collected.

### 2.3. Determination of Stroke or Stroke-Mimic

The diagnoses of the patients were determined by the criteria used by Hand et al. [7]. The data were classified as a definite stroke/TIA, probable stroke/TIA, possible stroke/TIA, and definite nonstroke. A definite stroke is when history and examination are typical of a vascular event and there is supporting neuroimaging data. A definite TIA has resolution of symptoms within 24 hours and no neuroimaging sequela. A probable stroke was when clinical features suggest a vascular etiology, but neuroimaging does not support the claim, at the time of acquisition. Possible stroke had less convincing evidence of vascular etiology (e.g., encephalopathy) and another explanation is more reasonable. A definite nonstroke was when the clinical features did not suggest a vascular etiology, and supportive investigations convene to an alternate explanation (e.g., tumors). The data was then grouped as stroke (definite and probable stroke/TIA) or stroke-mimic (definite nonstroke and possible stroke/TIA). These diagnoses were determined by the initial assessment of the resident/fellow, staff, and then the final study reviewer. In case of a discrepancy, the diagnoses were reached by consensus. The kappa value calculated between the resident, fellow, staff, and the final study reviewer was moderate at 0.6 (63.4% agreement).

### 2.4. Statistical Analysis

Differences between the two groups (stroke versus stroke-mimic) were assessed with descriptive statistics. Chi-square (categorical variables), *t*-test (continuous), and binary multiple logistic regression (multivariate analysis) were used to compare strokes versus stroke-mimics. Odds ratios (OR) were calculated between the two groups (stroke versus mimic) with 95% confidence intervals for univariate analysis. SPSS (IBM Corporation, version 20.0, Armonk, NY) was used for statistical analyses.

## 3. Results

During the 12-month period, a total of 93 patients (average age 69.6 ± 12.9 years) were included in our study. After a panel review, 57 patients (mean age of 69.6 years, 57.9% female) were determined as having a stroke (definite stroke/TIA or probable stroke/TIA). Thirty-six patients (mean age of 69.5 years, 50% female) were identified as having a stroke-mimic (possible stroke/TIA or definite nonstrokes). The majority of patients were discharged home or to rehabilitation (86%). Baseline characteristics of the strokes and stroke-mimics are found in Table 1.

### 3.1. Circumstances of Acute Stroke Notifications

For all patients, the median NIHSS score was 10 (range of 0–41) with 34.0% having an NIHSS of ≤5. Ten (27.8%) of the stroke-mimics had NIHSS ≤5 compared to 42.1% of stroke patients. This difference was nonsignificant. The exact time of onset was determined in all patients with the average time of neurological evaluation from last normal being 26.5 ± 123.8 hours. The most common primary care team were cardiothoracic surgery (33%), closely followed by cardiology (29%). Eighteen patients (19.4%) presented with symptoms first noticed upon awakening.

It was noted that stroke patients were significantly more likely to be obese (*p* = 0.03), had a prior history of stroke (*p* = 0.05) and atrial fibrillation at the time of onset or within 72 hours of the stroke activation (*p* = 0.03), and had been admitted to the cardiology service (*p* = 0.01). A total of fifty-eight patients were admitted to the cardiovascular service (cardiothoracic surgery and cardiology). Of those with atrial fibrillation at the time of onset or within 72 hours of the stroke, seventeen were on antiplatelet (*n* = 14) or therapeutic anticoagulation (*n* = 3). Nine patients were considered subtherapeutic on anticoagulation. Five patients were never on antiplatelet or anticoagulation. Two had antiplatelet medications withdrawn during hospitalization. Additionally, the subjective complaint of a weak hand (*p* = 0.03, OR 6.24) or objective hemiparesis (*p* = 0.03, OR 3.6) was more likely a stroke. In contrast, if a consult was for subjective altered mental status, it was significantly more likely a stroke-mimic (*p* ≤ 0.0001; Tables 1 and 2).

There were no significant differences between the two groups in comparison of medications, laboratory values, vitals, state of consciousness, past surgical history, and past medical history of hypertension, hypercholesterolemia, coronary artery disease, diabetes mellitus, past tobacco abuse, peripheral vascular disease, migraine, epilepsy, cancer, prior heart surgery, or a family history of stroke.

### 3.2. Etiologies of Strokes and Stroke-Mimics

The majority of strokes were found to be cardioembolic (71.9%) compared to a large vessel occlusion (14.0%) or small vessel occlusion (3.5%; Table 3). The most common etiology of stroke-mimics was toxic/metabolic (38.9%) of which medications (opioid/benzo) were the most common source (28.6%; Table 4). Other common etiologies included seizure (22.2%) and syncope (13.9%). One consult was called for pain related to placement of a peripheral intravenous line.

### 3.3. Timing of the In-Hospital Strokes

Overall, the timing of evaluation from last known well was 436.8 ± 768.1 hours. Of the stroke patients, the majority (*n* = 29, 50.9%) were assessed within three hours of last known well. The average times to neurology evaluation, to CT, and to treatment were 35 min, 68 min, and 237 min, respectively (Table 5). The delay for in-hospital strokes was in obtaining the CT and then initiating the treatment.

## 4. Discussion

An efficient stroke alerting system for in-patients is an advancement that is continuously evolving. The clinical assessment of acute strokes remains the standard and largely determines therapy. However, determining a stroke versus a stroke-mimic can be difficult. This study highlights that of the 93 patients who were suspected of having an acute stroke at our institution, the majority (61.3%) were found to have a stroke with the remainder having a stroke-mimic.

Our findings are consistent with a study by Byrne et al., who reviewed 106 patients admitted to an in-patient stroke unit, and found that 78 (73.5%) of the patients had strokes or TIAs, and the remaining were stroke-mimics (26.4%) [5]. A similar study has reported the occurrence of in-hospital stroke-mimics to be 41% [12]. However, stroke-mimics may account for up to 63.4% of the acute stroke consults [13].

Recognition of acute strokes in a timely manner is a challenge [14]. Clinical scores to identify strokes have been created for the ED [15]. However, their utility in the in-hospital patients with neurological changes is limited given the significant comorbidities. Thus, collaborative educational initiatives among physicians and nursing to improve quality and timeliness of acute stroke consults are necessary.

Additionally, our study showed which predictor variables can delineate in-hospital strokes from stroke-mimics. The most significant predictors for a stroke were subjective complaint of a weak hand, objective hemiparesis, points given on NIHSS 1b, a normal mental status, being admitted on the cardiology service, having atrial fibrillation at the time of onset or during hospitalization, previous stroke history, and obesity, which agrees with previous estimates [12, 16]. On the contrary, when a consult was called for altered mental status, it was most likely a stroke-mimic.

From our study, an interesting distinguishing predictor of stroke from a stroke-mimic was atrial fibrillation at the time of the stroke or within 72 hours of the stroke. This finding may be a reflection of an institutional bias. Our institution has an active cardiac center (i.e., cardiology and cardiothoracic surgery) where 62.4% of the stroke alerts were activated. However, it is consistent with previous observations that patients with in-hospital strokes were more likely to have atrial fibrillation than out-of-hospital strokes [10, 11, 16–18]. Additionally, cardioembolic strokes are much more common in the in-hospital stroke population compared to the community strokes [11, 12, 19, 20]. 31.0% of the atrial fibrillation patients in our study were subtherapeutic on their anticoagulation with an additional 6.9% having their antithrombotics discontinued during hospitalization for surgical procedures. Indeed, withdrawal of antithrombotics and subtherapeutic anticoagulation are risks for ischemic stroke [20]. Previous studies have demonstrated that patients with cardioembolic strokes from atrial fibrillation have poorer prognosis [21]. Thus, early resumption of antithrombotics and/or therapeutic anticoagulation is imperative.

Stroke alerts for altered levels of consciousness are significantly more likely to be a stroke-mimic. This is consistent with the findings of Libman et al. [18] and an editorial by Benbadis et al. [22]. An in-hospital stroke study by El Husseini and Goldstein found similar results. They identified 93 in-hospital acute stroke consults and found that the consults for altered mental status were significantly likely a stroke-mimic with the most common diagnosis for a stroke-mimic ultimately being metabolic and/or infectious [13]. The commonest etiologies of stroke-mimics from our study were toxic/metabolic (especially benzos, opioids), seizures, syncope, and sepsis, which agrees with previous studies [8, 23].

A recent study evaluated the use of red cell distribution width (RDW) as a marker to differentiate a stroke from stroke-mimics in young patients and demonstrated that the mean RDW values of young patients with stroke (14.9 ± 1.2) were significantly higher than patients with stroke-mimics (epilepsy or MS) (13.3 ± 1.2, 13.4 ± 0.6, *p* < 0.0001, resp.) [24].

In general, the in-hospital stroke patients may be less likely to receive IV rt-PA, secondary to the longer time to neuroimaging [25]. Additionally, thrombolytic treatment may reflect the fact that in-patients having a neurological change have more complex medical comorbidities that must be taken into account during the evaluation, transportation to the CT scanner, and administration of thrombolytic therapy. Future studies should focus on improving time to CT and time from CT to treatment in the in-hospital setting and then evaluate treatment. Other diagnostic testing such as MRI and/or EEG in distinguishing a stroke from a stroke-mimic should not come at the cost of time in patients awaiting IV tPA [15].

Lastly, 42.1% of our stroke patients had NIHSS ≤5 compared to 27.8% of our stroke-mimics. This is an interesting observation since traditionally NIHSS correlates with stroke severity [26], and higher NIHSS scores are commonly associated with strokes [23]. Traditionally in-patient strokes have longer length of stay and worse prognosis [27]. However, in our study, stroke-mimics did not have a significant difference from the stroke population in discharged needs. Our data may represent the complexity of our patient population.

Several limitations are identified with the present study. Our study is a retrospective chart review of recognized strokes. It is possible that clinically silent or minor strokes may not have been recognized by medical personnel and, thus, would not be included in this study. Additionally, the majority of patients were admitted for cardiac intervention which biases the population to having cardioembolic strokes. An MRI of the brain was only available in 36.6% of the patients to confirm strokes. Lastly, we could not follow patients longitudinally to track outcomes.

## 5. Conclusion

In summary, the in-hospital stroke patients are a unique treatment group given their medical complexity which can pose challenges in acute stroke management. Differentiating stroke from stroke-mimic is crucial. Based on our study, a patient is more likely to have an acute ischemic stroke when presenting with a subjective complaint of a weak hand, objective hemiparesis, significant points on the NIHSS 1b, normal mental status, having atrial fibrillation at the time of onset or during hospitalization, past stroke history, and obesity.

## Figures and Tables

**Table 1:** Patient characteristics of strokes and stroke-mimics.

	All patients		Stroke patients only		Mimics only	
	*n* = 93		*n* = 57		*n* = 36	
Age, y, mean ± SD	69.6 ± 12.9		69.6 ± 13.8		69.5 ± 11.7	
Male, *n* (%)	42	45.2	24	42.1	18	50.0
Hrs from onset, mean ± SD	26.5 ± 123.8		38.2 ± 157.2		8.02 ± 15.0	
*Past history/stroke risk factors:*						
HTN, *n* (%)	64	68.8	37	64.9	27	75
Diabetes mellitus, *n* (%)	23	24.7	13	22.8	10	27.8
Hypercholesterolemia, *n* (%)	43	46.2	22	38.6	21	58.3
Atrial fibrillation, *n* (%)	39	41.9	29	50.9	10	27.8^*∗*^
CAD, *n* (%)	48	51.6	28	49.1	20	55.6
Past tobacco abuse, *n* (%)	44	47.3	24	42.1	20	55.6
Cancer, *n* (%)	15	16.1	7	12.3	8	22.2
Prior stroke, *n* (%)	19	20.4	8	14	11	30.6^*∗*^
Prior heart surgery, *n* (%)	38	40.9	24	42.1	14	38.9
Obesity, *n* (%)	15	16.1	13	22.8	2	5.6^*∗*^
Family history of stroke, *n* (%)	17	18.3	10	17.5	7	19.4
*Presentation:*						
Awoke from sleep, *n* (%)	18	19.4	13	22.8	5	13.9
Coma, *n* (%)	10	10.8	6	10.5	4	11.1
Altered mental status, *n* (%)	33	35.3	12	21.1	21	58.3^*∗∗*^
Weak hand, *n* (%)	15	16.1	13	22.8	2	5.6^*∗*^
Hemiparesis, *n* (%)	28	30.1	22	38.6	6	16.7^*∗*^
AMET/CMET involved, *n* (%)	6	6.45	4	7.02	2	6
2CLOT activation by non-MD provider, *n* (%)	59	63.4	37	64.9	22	61.1
Cardiology, *n* (%)	27	29	22	38.6	5	13.9^*∗*^
Cardiothoracic surgery service, *n* (%)	31	33.3	19	33.3	12	33.3
*Medication:*						
Antiplatelet, *n* (%)	59	63.4	35	61.4	24	66.7
Anticoagulation, *n* (%)	23	24.7	16	28.1	7	19.4
Statin, *n* (%)	35	37.6	20	35.1	15	41.7
*Examination:*						
SBP, mean ± SD	128 ± 26.6		131 ± 27.2		123 ± 25.1	
Pulse, mean ± SD	82.3 ± 18.4		84.2 ± 18.8		79.3 ± 17.5	
Temperature (Celsius), mean ± SD	36.5 ± 0.72		36.6 ± 0.75		36.2 ± 0.59	
Baseline NIHSS, median (range)	10	0–41	10	0–41	11.5	0–35
NIHSS ≤ 5, *n* (%)	34	36.6	24	42.1	10	27.8
MRS 0 to 2, *n* (%)	66	71	41	71.9	25	69.4
*Laboratory/imaging:*						
WBC, K/uL, mean ± SD	10.5 ± 7.41		9.78 ± 3.78		11.7 ± 10.9	
Platelets, K/uL, mean ± SD	177 ± 85.6		178 ± 93.5		175 ± 72.7	
Sodium, mmol/L, mean ± SD	137 ± 4.32		138 ± 3.49		136 ± 5.29	
BUN, mg/dL, mean ± SD	27.7 ± 18.8		26.7 ± 17.7		29.3 ± 20.6	
Creatinine, mg/dL, mean ± SD	1.51 ± 1.19		1.43 ± 1.02		1.64 ± 1.42	
Glucose, mg/dL, mean ± SD	123 ± 45.5		119 ± 33.5		129 ± 59.9	
Brain MRI, *n* (%)	34	36.6	23	40.4	11	30.6
*Discharged:*						
Home, *n* (%)	40	43	23	40.4	17	47.2
Rehabilitation, *n* (%)	40	43	25	43.9	15	41.7
Death, *n* (%)	13	14	9	15.8	4	11.1

^*∗*^ *p* < 0.05 ;  ^*∗∗*^ *p* < 0.0001.

CAD, coronary artery disease; HTN, hypertension; 2CLOT, in-hospital stroke alerting system, AMET/CMET, in-hospital code blue team; NIHSS, NIH Stroke Scale; MRS, Modified Rankin Scale.

**Table 2:** Multivariate analysis.

Variables	OR	*p*
Hemiparesis	3.6	0.042
NIHSS item 1b	0.46	0.17
Normal mental status	0.28	0.34
Subjective complaint of weak hand	6.24	0.042

NIHSS, National Institute of Health Stroke Scale; OR, odds ratio.

**Table 3:** Stroke mechanisms.

Stroke mechanisms	*n*	%
Cardioembolic	41	71.9
Large vessel	8	14.0
Small vessel	2	3.5
Other etiologies	4	7.0
Cryptogenic	2	3.5

*n*, number.

**Table 4:** Etiologies of stroke-mimics.

Stroke-mimics	*n*	%
Toxic/metabolic^*∗*^	14	38.9
Seizure	8	22.2
Syncope	5	13.9
Sepsis	4	11.1
Tumor	1	2.8
Peripheral neuropathy	1	2.8
Functional	1	2.8
Dementia	1	2.8
Spinal cord lesion	1	2.8
Vestibular	0	0.0
Migraine	0	0.0

^*∗*^Toxic metabolic causes	*n*	%

Medication (opioid/benzo)	4	28.6
Hyponatremia	2	14.3
Pulmonary distress	2	14.3
Hepatic failure	2	14.3
Hyperglycemia	1	7.1
Renal/hyperuricemia	1	7.1
Hyperkalemia	1	7.1
Attempting IV line	1	7.1

IV, intravenous; benzo, benzodiazepine; *n*, number.

**Table 5:** In-hospital stroke times.

Treated in-hospital strokes	Minutes	StDev
Stroke onset to neuro evaluation	34.8	21.4
Stroke onset to treatment	236.6	65.1
Stroke onset to CT	67.6	27.7
Neuro evaluation to CT	32.8	10.4
Neuro evaluation to needle	213.8	83.7

StDev, standard deviation; neuro, neurological; CT, computed tomography.
